# Multilevel comparison of deep learning models for function quantification in cardiovascular magnetic resonance: On the redundancy of architectural variations

**DOI:** 10.3389/fcvm.2023.1118499

**Published:** 2023-04-18

**Authors:** Clemens Ammann, Thomas Hadler, Jan Gröschel, Christoph Kolbitsch, Jeanette Schulz-Menger

**Affiliations:** ^1^ Working Group on CMR, Experimental and Clinical Research Center, A cooperation between the Max Delbrück Center for Molecular Medicine in the Helmholtz Association and Charité — Universitätsmedizin Berlin, Berlin, Germany; ^2^ Charité — Universitätsmedizin Berlin, corporate member of Freie Universität Berlin and Humboldt-Universität zu Berlin, Berlin, Germany; ^3^ Max Delbrück Center for Molecular Medicine in the Helmholtz Association (MDC), Berlin, Germany; ^4^ DZHK (German Centre for Cardiovascular Research), partner site Berlin, Berlin, Germany; ^5^ Physikalisch-Technische Bundesanstalt (PTB), Braunschweig and Berlin, Germany; ^6^ Department of Cardiology and Nephrology, HELIOS Hospital Berlin-Buch, Berlin, Germany

**Keywords:** cardiovascular magnetic resonance, MRI, artificial intelligence, deep learning, cardiac image segmentation, cardiac function quantification, quality control

## Abstract

**Background:**

Cardiac function quantification in cardiovascular magnetic resonance requires precise contouring of the heart chambers. This time-consuming task is increasingly being addressed by a plethora of ever more complex deep learning methods. However, only a small fraction of these have made their way from academia into clinical practice. In the quality assessment and control of medical artificial intelligence, the opaque reasoning and associated distinctive errors of neural networks meet an extraordinarily low tolerance for failure.

**Aim:**

The aim of this study is a multilevel analysis and comparison of the performance of three popular convolutional neural network (CNN) models for cardiac function quantification.

**Methods:**

U-Net, FCN, and MultiResUNet were trained for the segmentation of the left and right ventricles on short-axis cine images of 119 patients from clinical routine. The training pipeline and hyperparameters were kept constant to isolate the influence of network architecture. CNN performance was evaluated against expert segmentations for 29 test cases on contour level and in terms of quantitative clinical parameters. Multilevel analysis included breakdown of results by slice position, as well as visualization of segmentation deviations and linkage of volume differences to segmentation metrics *via* correlation plots for qualitative analysis.

**Results:**

All models showed strong correlation to the expert with respect to quantitative clinical parameters (*r_z_*_′_ = 0.978, 0.977, 0.978 for U-Net, FCN, MultiResUNet respectively). The MultiResUNet significantly underestimated ventricular volumes and left ventricular myocardial mass. Segmentation difficulties and failures clustered in basal and apical slices for all CNNs, with the largest volume differences in the basal slices (mean absolute error per slice: 4.2 ± 4.5 ml for basal, 0.9 ± 1.3 ml for midventricular, 0.9 ± 0.9 ml for apical slices). Results for the right ventricle had higher variance and more outliers compared to the left ventricle. Intraclass correlation for clinical parameters was excellent (≥0.91) among the CNNs.

**Conclusion:**

Modifications to CNN architecture were not critical to the quality of error for our dataset. Despite good overall agreement with the expert, errors accumulated in basal and apical slices for all models.

## 1. Introduction

Cardiovascular magnetic resonance (CMR) is considered the gold standard for an accurate and reproducible assessment of cardiac anatomy and function (1, 2). Furthermore, CMR is unique in noninvasive imaging for its capabilities to characterize myocardial tissue (3) and is increasingly being included in clinical guidelines (4–6). Quantitative clinical parameters for ventricular function such as end-diastolic and end-systolic volumes, ejection fraction and left ventricular myocardial mass are predictive of patient outcome and relevant for treatment (6). Their calculation depends on exact contouring of ventricular blood volumes and myocardium. Manual segmentation is time-consuming and typically takes trained physicians up to 20 min per subject (7).

In recent years convolutional neural networks (CNN) demonstrated promising results for automating semantic segmentation tasks in the medical domain (8, 9). Next to a substantial time advantage, the reproducibility of automatic image analysis eliminates the interobserver error between different readers and the intraobserver variability for the same reader at different times. Deep learning-based methods are easy to deploy to medical image segmentation tasks as they do not require geometric a-priori-knowledge or extensive feature engineering.

Automated deep learning approaches match or exceed the performance of established conventional algorithms, typically measured by total segmentation overlap and mean differences in clinical parameters. Despite published overall results in the range of interobserver errors for cardiac function quantification (7, 10, 11), however, CNNs continue to make errors that compromise their acceptance for clinical application (10, 12) as generalizability and reliability remain challenging (13). Errors are not necessarily reflected in the overall results of the method, but they violate anatomical principles and are incomprehensible to human experts. Variations to the U-Net architecture [e.g., residual connections (14) or inception modules (15)] intend to improve robustness and accuracy. Yet, it remains questionable to what extent these modifications offer a substantial benefit to the segmentation accuracy given the increasing complexity and computational power requirements.

The aim of this work is to provide a detailed analysis and comparison of three different CNN architectures for the quantification of ventricular function in short-axis cine images.

## 2. Material and methods

Ethical approval for this retrospective study was obtained from the ethics committee of Charité — Universitätsmedizin Berlin (approval number EA1/367/20). Part of this work has been presented at the scientific sessions of the 2022 Joint Annual Meeting ISMRM-ESMRMB & ISMRT 31st Annual Meeting (16).

### 2.1. Data

The dataset consists of routine clinical magnetic resonance studies of 148 patients randomly split into 119 training cases (1,955 images) and 29 test cases (479 images). Full specification on the dataset is published by Gröschel et al. (17). Seven patients were excluded due to technical limitations. Indications for CMR include coronary artery disease, cardiomyopathies, myocarditis, valvular heart disease, and cardiac mass. As a result, cardiac function in the study population spans the full clinical range of left ventricular ejection fraction from 12% to 78%. Short-axis cine images were acquired on a 1.5 Tesla scanner (MAGNETOM Avanto Fit, Siemens Healthineers, Erlangen, Germany) using a prototype 2-shot 2D cine Compressed Sensing balanced steady-state free precession sequence. Each short-axis stack contains a series of images sliced from the apex to the atrioventricular junction (7 mm slice thickness, no gap) over 25 phases in the cardiac cycle. The 2-shot sequence acquires one slice in two cardiac cycles (plus one additional cardiac cycle for preparation) per breath hold, providing an acceleration factor of 5.6.

A trained physician manually segmented the left ventricular (LV) endocardial and epicardial borders as well as the right ventricular (RV) endocardial border in end-diastole and end-systole using dedicated software (cvi42 version 5.6.2, Circle Cardiovascular Imaging, Calgary, Canada) according to the post-processing consensus statement by the Society for Cardiovascular Magnetic Resonance (1). The endocardial contour encloses the ventricular blood pool for the calculation of volumes and, together with the epicardial contour, delimits the myocardium. Papillary muscles and trabeculae were included in blood pool volumes, and not added to the myocardial mass.

### 2.2. CNN models

**Figure 1:**
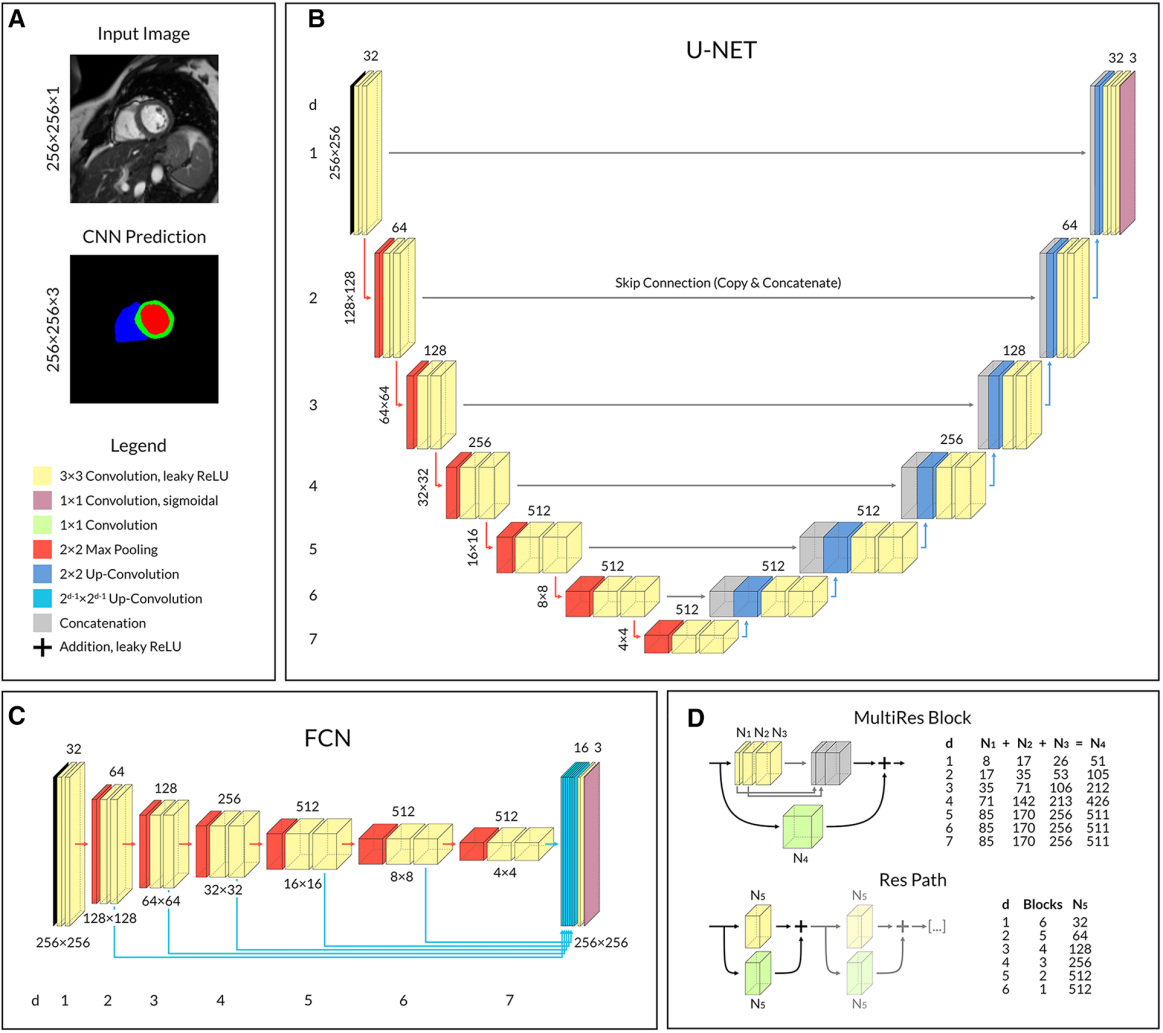
Convolutional neural network architectures. (**A**) Network input and output example with corresponding shape. Shared legend for operational blocks. (**B**) U-Net with symmetrical encoder and decoder architecture. (**C**) FCN with one-step decoding by 16-channel up-convolution and concatenation from each layer. (**D**) In MultiResUNet, a single MultiRes Block replaces the two convolutional operations in each layer of U-Net, and a Res Path replaces each skip connection. The number of feature channels varies with the depth of the layer in the CNN and is given in the two tables on the right. Legend: ReLU, rectifier linear unit; *d*, layer depth; *N*_1–5_, Number of feature channels according to table.

Three different published CNN architectures were compared: U-Net (18), a variant of Fully Convolutional Network (FCN) (19) as described by Xie and Tu (20), and MultiResUNet (21). U-Net has established itself as the reference model for deep learning in medical image segmentation and is heading the leaderboards of recent challenges (10, 22). The FCN architecture was selected due to its popularity (8) and use in major publications in the field (7). MultiResUNet is a more recent CNN variation and was chosen because it promises more stable results on challenging images (21, 23), which has proven to be a major problem with other architectures (10, 13). In the convolutional layers of CNNs the input of each neuron is computed as the dot product with a small learned convolutional matrix. The input image is gradually encoded into a low-resolution, feature-rich latent space, which must then be up-sampled to original resolution to eventually perform a pixel-wise classification (24). The U-Net is a special type of FCN that uses a symmetrical encoder and decoder structure (Figure 1B). Furthermore, spatial information from the down-sampling pathway is propagated to the up-sampling part through concatenation *via* skip connections. The FCN, on the other hand, has only one decoding layer, so that predictions from the different encoding layers are fused and up-sampled to the original resolution in one step (Figure 1C). The MultiResUNet is based on the U-Net but uses more complex convolutional layers with the intention of making segmentation results more robust to outliers. It embraces the idea of inception modules and residual learning. Each convolutional layer in the MultiResUNet consists of three successive convolutional operations (having the same effect as different kernel sizes) that are concatenated and to which a residual connection is added (Figure 1D MultiRes Block). The modified skip connections involve a variable sequence of convolutional steps, each with a residual connection (Figure 1D Res Path).

Our implementations of the three network architectures share a comparable number of trainable parameters and similar hyperparameters. Input to the networks was a normalized image with shape 256 × 256 × 1; the output was a segmentation map with shape 256 × 256 × 3 for LV blood pool, RV blood pool and left ventricular myocardium respectively (Figure 1A). We aimed for a minimum feature map size of 4 × 4 in the deepest layer of the encoding path to ensure good context aggregation, as suggested by Isensee et al. (25). The number of filter kernels was doubled for each convolutional block during encoding, however limited to a maximum of 512 to counteract the exponential growth in the number of parameters. Convolutional layers used leaky rectifier linear unit activation functions followed by batch normalizations except for the output layer, which used a sigmoidal activation function. Dropout with a rate of 0.1 was added to each convolutional block for the U-Net and FCN.

### 2.3. Training pipeline

Neural network performance is significantly affected by data pre-processing and training pipeline design. We used the exact same framework for all networks to make the architectures comparable, starting with a conversion of manual contour points to Shapely (26) polygon objects and mapping them to the respective images. Images and contours were resized by a factor of 1.5 before applying extensive random data augmentation using the imgaug (27) library including affine transformation, zooming, average pooling, Gaussian noise and blurring as well as contrast and brightness alterations. Image augmentation parameters were defined based on published configurations (25) and adjusted to produce profound but not extreme alterations so that cardiac structures were not truncated and remained visually delineable. Only after image augmentation, the ground truth contours were rasterized to segmentation maps to preserve subpixel information that would have otherwise been lost in the floating-point operations during preprocessing. In clinical practice interpolated images are commonly used for segmentation tasks to draw contours with subpixel resolution resulting in non-integer contour definitions.

The segmentation networks and deep learning were implemented in Python 3.8.10 with TensorFlow 2.8.0 (28). The CNNs were trained with a batch size of eight images for a maximum of 700 epochs depending upon an early stopping mechanism safeguarding an increasing Dice similarity coefficient (Dice, see 2.3) within 50 epochs. Training time per epoch (1,960 images) was 95s for U-Net, 97s for FCN and 161s for MultiResUNet on a workstation with NVIDIA Tesla P100 16GB and Quadro P4000 GPU, 24-core 3.40 GHz Intel Xeon CPU and 512 GB of RAM. Adam algorithm was used to optimize a combined binary cross-entropy and Dice loss function with polynomial decaying learning rates starting at 0.01.

$$\eta =0.01\;*\;{\left(1-\frac{t}{700}\right)}^{0.9}$$

Postprocessing was limited to extracting the largest polygon for each segmentation mask, vectorization to contours and back-transformation to original resolution.

### 2.4. Metrics

The evaluation of segmentation quality was based on quantitative clinical parameters and geometric segmentation metrics as well as visual inspection for qualitative analysis. Clinical parameters include end-diastolic and end-systolic volumes for the left ventricle (LVEDV, LVESV) and right ventricle (RVEDV, RVESV), the left and right ventricular ejection fraction (LVEF, RVEF), and the left ventricular myocardium (LVM). For better comparability with other work and in accordance with clinical practice all values for LVM are given for the end-diastole.

For segmentation metrics, the Dice similarity coefficient and the Hausdorff distance (Hd) were used to calculate the percentage overlap and the maximal regional distance respectively, the combination of which allows for the evaluation of geometrical differences between two individual segmentations *A* and *B*.

$$\begin{array}{rl} & \mathrm{Dice}\;(A,B)=2\;*\;\frac{\left|A\cap B\right|}{\left|A|+|B\right|}\;\\ & Hd\;(A,B)=\max\left(\underset{a\in A}{\sup}\;\underset{b\in B}{\inf}\;d(a,b),\underset{b\in B}{\sup}\;\underset{a\in A}{\inf}\;d(a,b)\right) \end{array}$$

Average Dice values are significantly influenced by slices not segmented in *A* and *B* resulting in a perfect value of 100%, but also by slices segmented by only one of the methods resulting in a value of zero percent. Therefore, two Dice metric averages were computed, one for all slices and thus considering segmentation decisions (whether a segmentation is given for the respective structure in each image) and the other only for images segmented by both, the expert and neural network. Especially the zero values distort Dice distribution and complicate the interpretation, which is why no standard deviation is given for the average Dice metric for all slices. CNN training required the calculation of a pixel-based Dice metric in the loss function, while exact Dice scores based on contours are reported in the analysis.

Binary metrics were used to gauge segmentation decision accuracy compared to the expert. In this context, precision (in medical science better known as the positive predictive value) describes the proportion of correctly considered slices among all slices segmented by a CNN for a corresponding structure. Recall, on the other hand, measures the percentage of correctly considered slices of all slices that should have been segmented and is commonly referred to in medicine as the sensitivity.

$$\mathrm{Precision}=\frac{t_{p}}{t_{p}+f_{p}};\;\mathrm{Recall}=\frac{t_{p}}{t_{p}+f_{n}}$$

*t*_p_: true positive, *f*_p_: false positive, *f*_n_: false negative.

### 2.5. Analysis

Network predictions for the 29 test cases were evaluated against the expert segmentations using the recently published dedicated software Lazy Luna (29). Analyses were performed on image (segmentation metrics) and patient (clinical parameters) levels using contours and not pixel-masks for all calculations. Quantitative clinical parameters were automatically calculated and linked to segmentation metrics *via* Lazy Luna, which allowed for a back-tracing of quantitative errors to segmentation differences illuminating their volumetric relevance.

Visualizations of segmentation errors facilitated manual qualitative analysis. Here, interactive correlation plots of Dice on one axis and volume difference in milliliters on the other provided an overview of the dataset and easy navigation for qualitative inspection. This, together with data tables and Bland-Altman plots in Lazy Luna, allowed difficult cases and outliers to be identified and the respective segmentation deviations to be visualized on the CMR images by clicking on the data points. The scatterplots were designed in such a way that segmentation decision errors and non-overlapping segmentations accumulate at the base with a Dice of zero. Slices that were neither segmented by the network nor the expert as well as perfectly matching segmentations, both have a Dice of 100 and no volume difference. These correspond to the top center data points.

**Figure 2:**
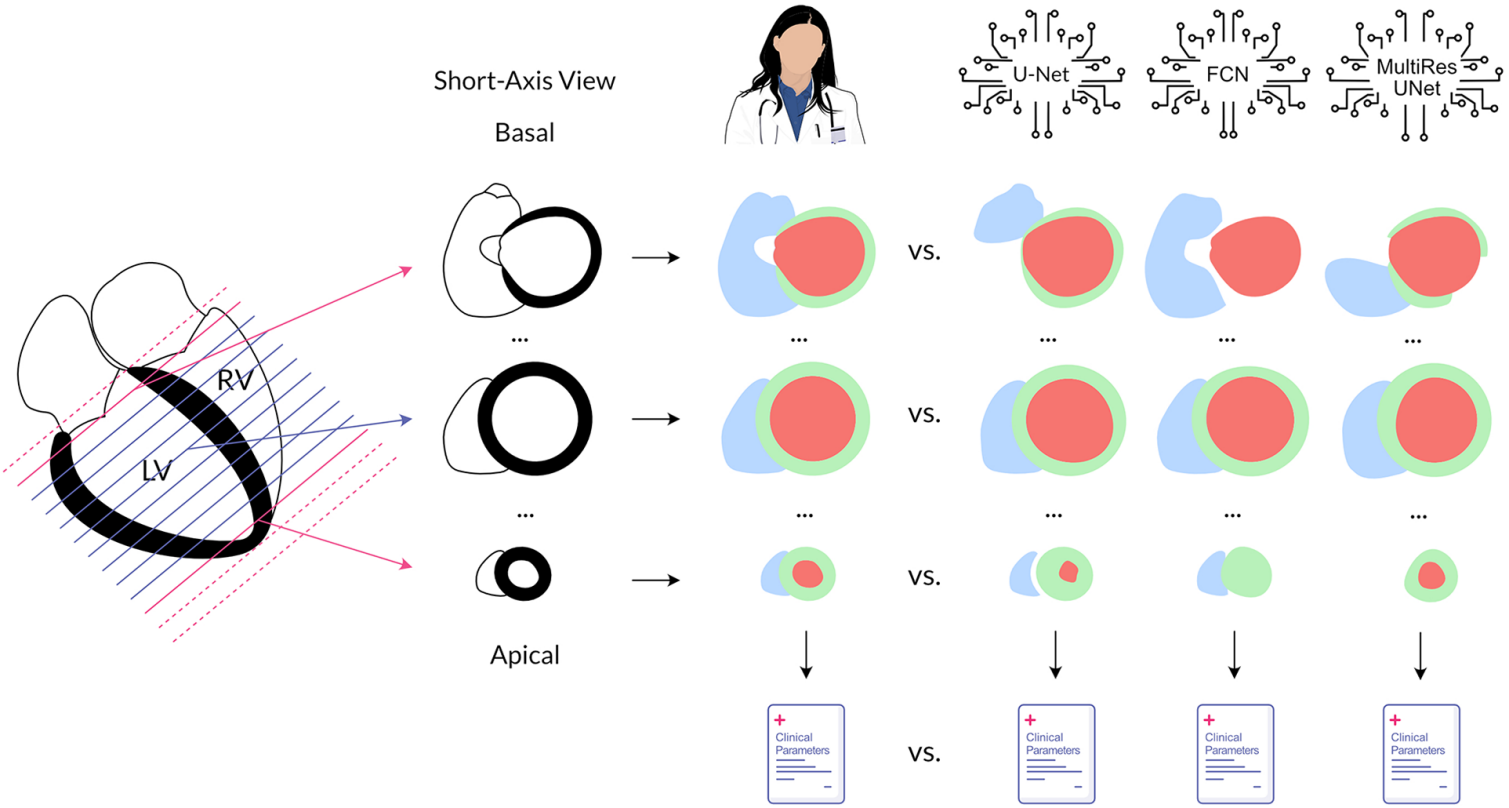
Method overview. Cardiovascular magnetic resonance cine images in the short axis were annotated by an experienced physician and three convolutional neural networks to quantify left and right ventricular function. The obtained segmentations and the clinical parameters calculated from them were compared for each neural network against the expert. In addition, analysis was performed separately by position in the heart. Legend: LV, left ventricle; RV, right ventricle; red, left ventricular cavity; green, left ventricular myocardium; blue, right ventricular cavity.

To evaluate the origin of quantitative errors, each image stack was subdivided by slice position in the heart (Figure 2). The most basal slice segmented by the expert and all slices incorrectly segmented by the respective CNN above it were defined as basal. Accordingly, the most apical slice segmented by the expert and all slices incorrectly segmented by the respective CNN below it were defined as apical. All other slices in between were designated midventricular. This allowed for a focused assessment of the typically difficult slices containing heart valves or apical trabeculation.

### 2.6. Statistics

Statistical analysis included the calculation of mean differences ± standard deviation for the quantitative clinical parameters and 2-sided paired *t*-tests to check for significant (significance level *α* = 0.05) deviations from the expert. The distribution of the errors for clinical parameters and the distribution of both Dice metrics were visualized using boxplots. Additionally, Pearson correlation coefficients were calculated for clinical parameters, and transformed to normalized values using Fisher's *z*′ to form overall correlation values. Dice and Hd as well as their means and standard deviations and precision and recall including their means were calculated for all CNNs compared to the expert segmentations. Segmentation metrics were additionally performed separately for basal, midventricular, and apical slices. Intraclass-correlation estimates among the three tested networks were calculated using R 4.2.2 based on a single-rating, consistency-agreement, 2-way mixed-effects model.

Lazy Luna was used for data preparation and calculation of segmentation metrics and clinical parameters. Statistical analysis and graphic creation were carried out in R 4.2.2 (using library psych 2.2.9), Python 3.8.10 (using packages SciPy 1.7.0, Matplotlib 3.4.3 and Seaborn 0.11.2) as well as Microsoft Excel for Mac 16.54.

## 3. Results

Mean combined processing and prediction time per test case (full image stack of 325–525 images) was 5.0s for U-Net, 4.6s for FCN and 7.3s for MultiResUNet.

### 3.1. Quantitative clinical results and segmentation accuracy

**Figure 3:**
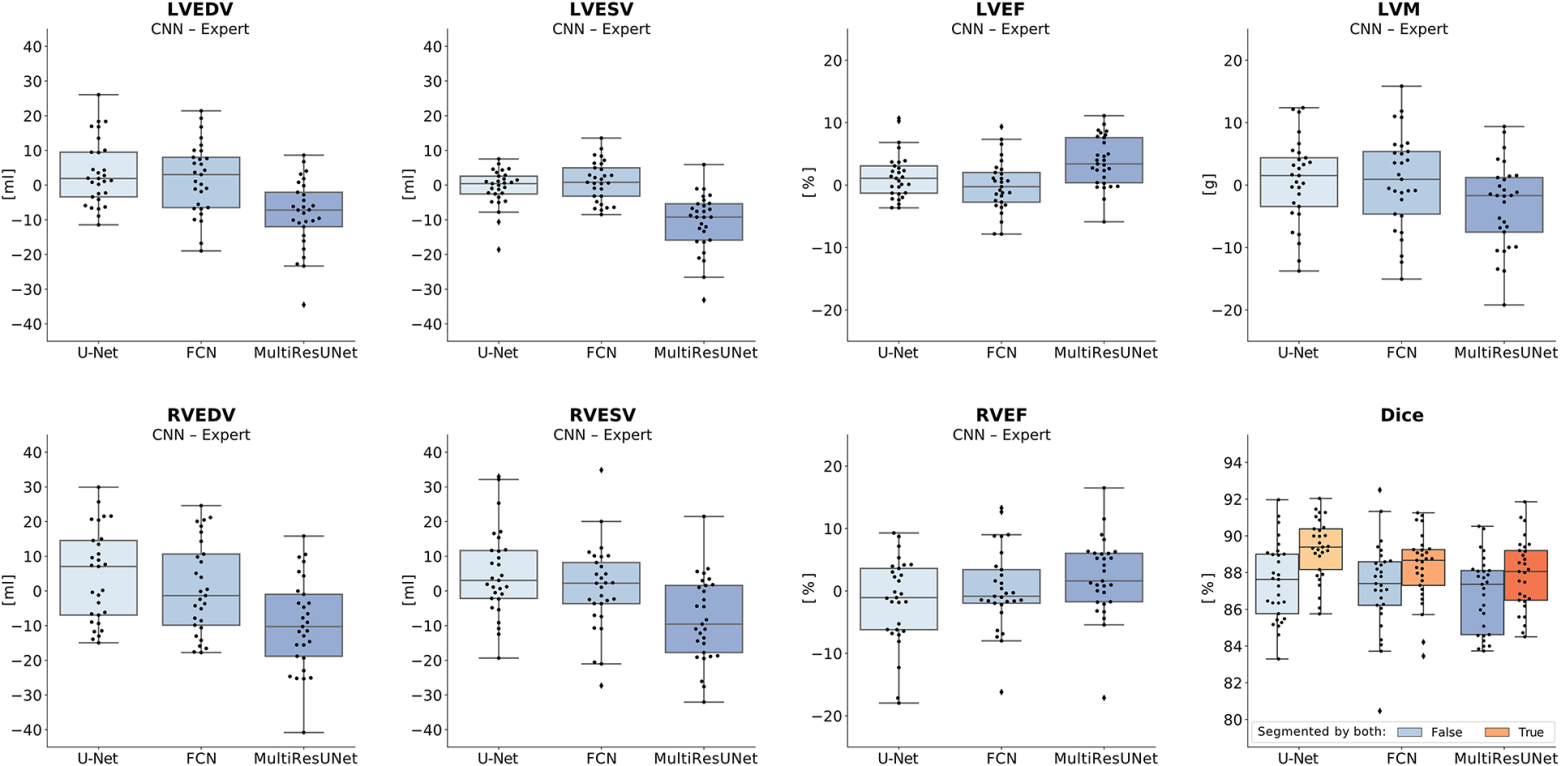
Candlelight plots of errors in clinical parameters and Dice values. Vertical boxplots show quantile one, median and quantile three of mean errors and Dice values for all test cases compared to the expert. The last graph displays two boxplots per network according to different definitions of the Dice metric: one for all images, another limited to images segmented by both expert and CNN. Legend: CNN, convolutional neural network; LVEDV, left ventricular end-diastolic volume; LVESV, left ventricular end-systolic volume; RVEDV, right ventricular end-diastolic volume; RVESV, right ventricular end-systolic volume; LVEF, left ventricular ejection fraction; RVEF, right ventricular ejection fraction; LVM, left ventricular myocardial mass.

**Figure 4:**
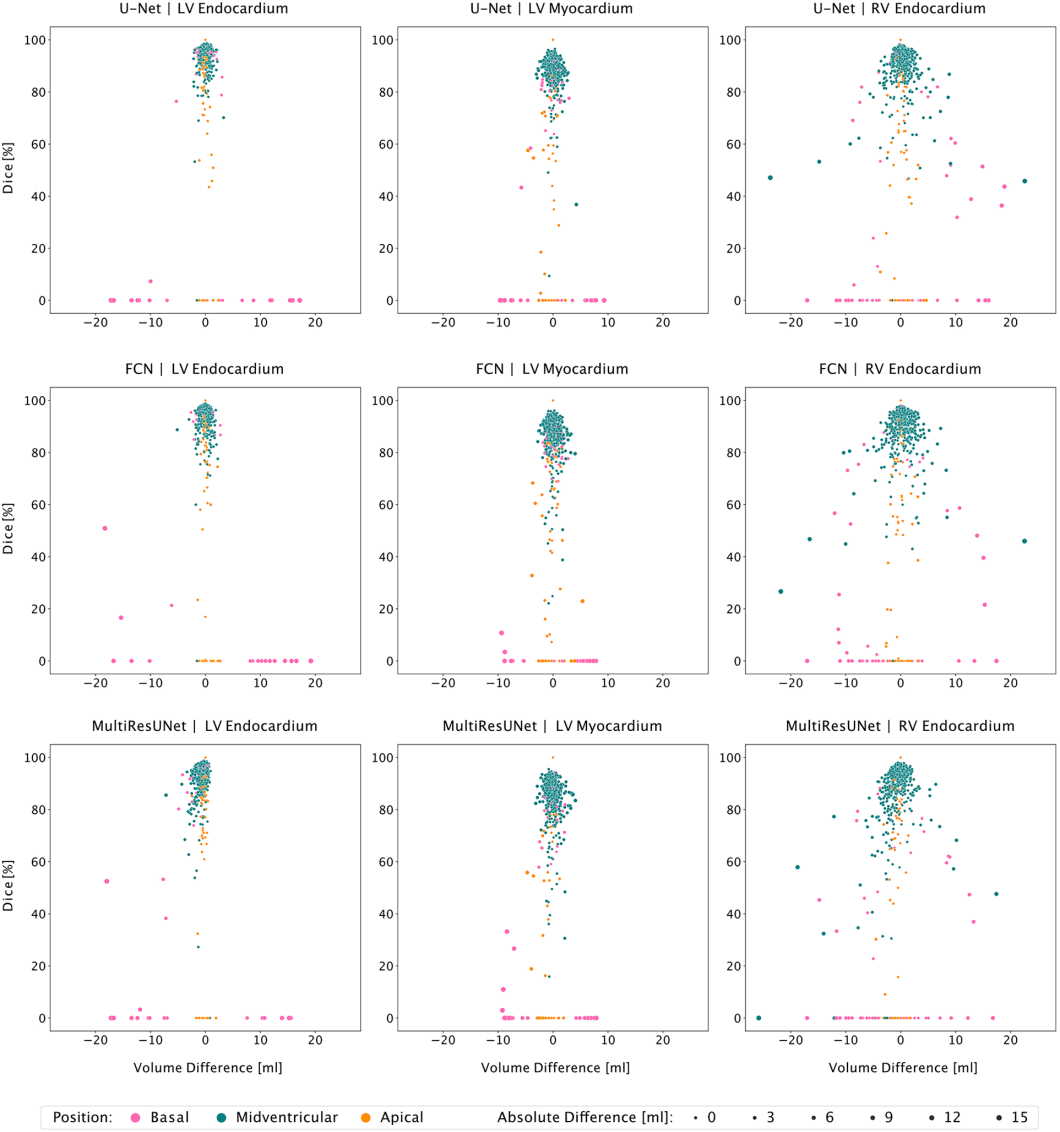
Correlation plots of segmentation comparisons according to slice position. Rows identify the network compared to the expert. Columns identify the considered contour type. Points represent contour comparisons characterized by volume difference and Dice value. Color implies the slice position.

All three networks showed strong correlations for left and right ventricular quantitative clinical parameters, as presented in Table 1 (average Pearson correlation *via* Fisher-*z*-transformation *r_z_*_′_ = 0.978, 0.977, 0.978 for U-Net, FCN, MultiResUNet respectively). The MultiResUNet significantly underestimated all volumes (LVEDV: *p* < 0.001; LVESV: *p* < 0.001; RVEDV: *p* < 0.001; RVESV: *p* = 0.001) and LVM (*p* = 0.02) and overestimated the LVEF (*p* < 0.001). The U-Net significantly overestimated LVEF (*p* = 0.05) and RVESV (*p* = 0.03). The distribution of the errors and the Dice values for the 29 test cases is illustrated in Figure 3 by candlelight boxplots. Dice values were consistently high (LV: 91.7%, 91.1%, 91.0%; LVM: 83.5%, 82.5%, 81.4%; RV: 85.1%, 85.8%, 84.9% for U-Net, FCN, and MultiResUNet respectively) and Hausdorff distances averaged within 1–2 voxels for LV and three voxels for RV, indicating good agreement between the segmentations of all three CNNs and the expert. The networks performed better for the left than for the right ventricle across all results in Table 1. Dice values were higher in end-diastole than in end-systole. Among all three CNNs, the right ventricle showed greater variance in the Dice metric for slices segmented by both methods (RVEDV: *σ* = 15.8% vs. LVEDV: *σ* = 8.6%; RVESV: *σ* = 19.7% vs. LVESV: *σ* = 9.2%) and in volume differences (RVEDV: *σ* = 13.3 ml vs. LVEDV: *σ* = 9.8 ml; RVESV: *σ* = 12.3 ml vs. LVESV: *σ* = 6.6 ml), as also demonstrated in Figure 4. Intraclass correlation (Table 2) was consistently excellent (≥0.91) for clinical parameters among U-Net, FCN and MultiResUNet. When estimated for all three CNNs and the expert, the intraclass correlation was good (0.85 for RVEF) to excellent (≥0.97 for all other parameters).

**Table 1:** Evaluation of CNNs on clinical parameters, segmentation metrics and segmentation decision metrics. Best Results Underlaid in Blue.

	Left Ventricle			Right Ventricle		
	U-Net mean (±*σ*)	FCN mean (±*σ*)	MultiResUNet mean (±*σ*)	U-Net mean (±*σ*)	FCN mean (±*σ*)	MultiResUNet mean (±*σ*)
EF (CNN—expert) [%]	1.4 ± 3.7*	−0.1 ± 4.1	3.7 ± 4.0*	−1.6 ± 6.9	0.3 ± 6.4	2.2 ± 6.2
Correlation	0.969	0.961	0.966	0.751	0.803	0.808
EDV (CNN—expert) [ml]	3.6 ± 9.5	1.9 ± 10.2	−8.2 ± 9.8*	4.3 ± 13.4	0.9 ± 13.3	−9.5 ± 13.2*
Correlation	0.994	0.993	0.995	0.962	0.963	0.963
Dice (all slices) [%]	92.2	91.3	91.8	86.9	86.7	85.8
Dice (slices segmented by both) [%]	95.4 ± 6.4	94.3 ± 10.2	94.1 ± 8.7	88.9 ± 15.3	87.5 ± 16.8	87.4 ± 15.4
Hd [mm]	1.9 ± 1.1	2.2 ± 1.8	2.3 ± 1.7	4.8 ± 5.9	5.2 ± 6.6	5.1 ± 6.1
ESV (CNN—expert) [ml]	−0.7 ± 5.3	1.1 ± 5.8	−10.6 ± 8.3*	5.1 ± 12.4*	0.8 ± 12.5	−8.2 ± 12.1*
Correlation	0.998	0.997	0.996	0.957	0.955	0.950
Dice (all slices) [%]	91.1	90.9	90.2	83.2	85.0	84.1
Dice (slices segmented by both) [%]	91.5 ± 9.1	91.3 ± 9.1	90.2 ± 9.5	82.6 ± 18.9	82.1 ± 20.4	82.5 ± 19.9
Hd [mm]	2.5 ± 1.7	2.5 ± 1.3	2.8 ± 2.0	6.0 ± 6.8	5.9 ± 6.3	6.0 ± 7.1
LVM (CNN—expert, in ED) [g]	0.7 ± 6.8	0.7 ± 7.6	−3.2 ± 6.9*			
Correlation	0.988	0.985	0.990			
Dice (all slices) [%]	83.5	82.5	81.4			
Dice (slices segmented by both) [%]	85.8 ± 9.9	84.0 ± 13.0	82.5 ± 12.9			
Hd [mm]	2.3 ± 2.1	2.6 ± 2.9	2.7 ± 2.9			

CNN, convolutional neural network; EF, ejection fraction; EDV, end-diastolic volume; ESV, end-systolic volume; LVM, left ventricular myocardial mass; Hd, Hausdorff distance; ED, end-diastole.

* *p* < 0.05.

**Table 2:** Intraclass correlation.

	CNNs ICC(3,1) [95% CI]	CNNs + Expert ICC(3,1) [95% CI]
LVEF	0.98 [0.96–0.99]	0.97 [0.95–0.98]
LVEDV	1.00 [0.99–1.00]	1.00 [0.99–1.00]
LVESV	1.00 [0.99–1.00]	1.00 [0.99–1.00]
LVM	1.00 [0.99–1.00]	0.99 [0.98–1.00]
RVEF	0.91 [0.85–0.95]	0.85 [0.76–0.92]
RVEDV	0.98 [0.97–0.99]	0.97 [0.95–0.99]
RVESV	0.98 [0.96–0.99]	0.97 [0.94–0.98]

ICC(3,1), 2-way mixed-effects, single-rater intraclass correlation; CI, confidence interval; LVEF, left ventricular ejection fraction; LVEDV, left ventricular end-diastolic volume; LVESV, left ventricular end-systolic volume; LVM, left ventricular myocardial mass; RVEF, right ventricular ejection fraction; RVEDV, right ventricular end-diastolic volume; RVESV, right ventricular end-systolic volume.

### 3.2. Multilevel analysis of error

**Figure 5:**
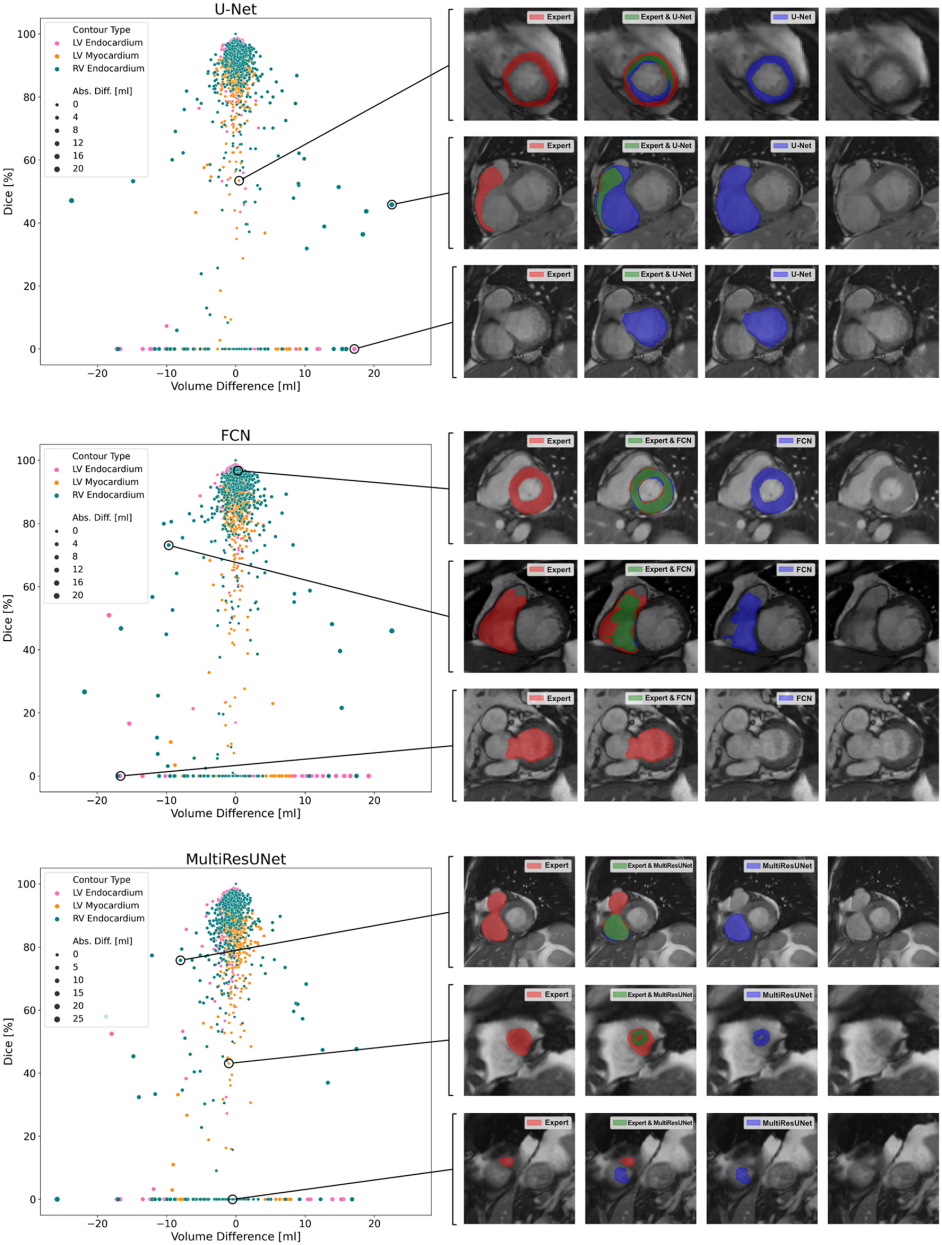
Correlation plots of segmentation comparisons. Each subplot shows the comparison of the contours of a neural network to the expert. Points represent contour comparisons and are distributed based on volume difference in milliliters and Dice. For interactive qualitative error analysis, visualizations of segmentation agreement could be displayed when clicking on a point. Examples are given on the right side of the figure.

The average Dice similarity coefficients for slices segmented by both CNN and expert were consistently higher than the ones for all slices (Figure 3). In Table 3, metrics for segmentation decision and quality according to slice position in the heart are examined and compared to the average absolute volume difference per slice. Here, the CNNs performed similarly, revealing difficulties in basal and apical slices. The overall results by slice position are summarized in Table 4 for all networks and across all contour entities. Dice values were low in basal (51.7%) and apical (43.9%) slices and segmentation decision errors were more frequent (mean precision: 80.6%, 99.7%, 72.4%; mean recall: 80.6%, 100.0%, 86.9% for basal, midventricular, apical respectively). The basal slices of the right ventricle showed exceptionally poor precision (68.4%), low Dice values (33.4%) and large average Hausdorff distances (19.1 ± 13.7 mm). In more than 40% of apical slices, the CNNs predicted LVM segmentations in which the expert had not segmented the left ventricular myocardium (average precision = 58.6%). The correlation plots in Figure 4 are color-coded according to slice position and subdivided by network and contour entity. Consistent with the results in Table 3, there is little scatter in the midventricular slices, with relevant volume differences in the basal slices. Apical slices showed low Dice values, but at the same time had negligible volume effects (mean absolute error per slice: 4.2 ± 4.5 ml for basal, 0.9 ± 1.3 ml for midventricular, 0.9 ± 0.9 ml for apical slices). In challenging test slices, repeated errors were made by all networks (Figure 5). The network architecture did not affect the type or quality of the segmentation errors, so it was not possible to infer a CNN from specific errors during visual inspection.

**Table 3:** Comparison of convolutional neural networks by segmentation metrics subdivided by contour entity and slice position.

		LV Endocardial Contour			LV Myocardial Contour			RV Endocardial Contour		
		U-Net mean (±σ)	FCN mean (±σ)	MultiResUNet mean (±σ)	U-Net mean (±σ)	FCN mean (±σ)	MultiResUNet mean (±σ)	U-Net mean (±σ)	FCN mean (±σ)	MultiResUNet mean (±σ)
Basal	Precision [%]	86.2	94.8	84.5	86.2	89.7	79.3	74.1	65.5	65.5
	Recall [%]	82.0	78.6	87.5	75.8	74.3	85.2	76.8	80.9	84.4
	Dice (all slices) [%]	67.1	68.7	67.0	55.3	53.1	54.2	35.2	33.5	31.6
	Dice (segmented by both) [%]	92.6 ± 13.0	91.2 ± 15.6	88.9 ± 17.3	81.8 ± 10.0	77.7 ± 21.9	77.7 ± 13.0	58.1 ± 37.3	59.1 ± 37.1	54.1 ± 36.5
	Hd [mm]	3.4 ± 3.3	3.8 ± 4.1	4.6 ± 4.8	5.1 ± 6.2	6.3 ± 9.1	6.1 ± 9.2	18.2 ± 13.4	18.3 ± 14.5	20.7 ± 13.2
	Abs. vol. diff. (per slice) [ml]	3.7 ± 5.2	3.7 ± 5.5	4.5 ± 5.5	2.8 ± 3.0	2.7 ± 3.1	3.0 ± 3.0	5.7 ± 5.0	5.7 ± 4.8	5.8 ± 4.3
Mid.	Precision [%]	99.8	99.8	100.0	99.7	100.0	99.7	100.0	99.8	98.8
	Recall [%]	100.0	100.0	100.0	100.0	100.0	100.0	100.0	100.0	100.0
	Dice (all slices) [%]	94.5	94.3	93.4	86.9	86.0	84.0	90.1	89.3	87.8
	Dice (segmented by both) [%]	94.6 ± 5.8	94.5 ± 4.7	93.4 ± 7.2	87.2 ± 7.8	86.0 ± 9.3	84.3 ± 9.8	90.1 ± 9.1	89.5 ± 9.8	88.9 ± 10.7
	Hd [mm]	2.0 ± 1.1	2.2 ± 1.0	2.4 ± 1.3	2.0 ± 1.0	2.1 ± 0.9	2.4 ± 1.4	4.2 ± 4.1	4.5 ± 4.3	4.4 ± 4.2
	Abs. vol. diff. (per slice) [ml]	0.5 ± 0.4	0.5 ± 0.5	0.8 ± 0.7	0.6 ± 0.5	0.6 ± 0.5	0.7 ± 0.5	1.3 ± 2.0	1.4 ± 2.0	1.5 ± 2.2
Apical	Precision [%]	77.6	82.8	75.9	55.2	65.5	55.2	86.2	87.9	65.5
	Recall [%]	91.8	85.7	91.7	94.1	90.5	94.1	67.6	82.3	84.4
	Dice (all slices) [%]	58.8	54.8	57.1	34.0	35.6	28.2	41.8	44.6	39.8
	Dice (segmented by both) [%]	81.1 ± 13.5	75.3 ± 22.6	80.4 ± 11.8	63.8 ± 18.4	58.1 ± 21.7	52.8 ± 26.2	68.6 ± 21.4	60.3 ± 29.3	68.0 ± 22.8
	Hd [mm]	2.4 ± 1.3	2.8 ± 2.0	2.3 ± 1.2	3.8 ± 2.2	4.9 ± 3.3	5.2 ± 3.4	5.2 ± 3.1	6.1 ± 4.5	4.5 ± 3.4
	Abs. vol. diff. (per slice) [ml]	0.5 ± 0.4	0.5 ± 0.5	0.5 ± 0.5	1.0 ± 0.8	1.3 ± 1.3	1.2 ± 1.0	1.0 ± 1.0	1.0 ± 0.9	1.1 ± 0.9

Hd, Hausdorff distance; abs. vol. diff., absolute volume difference; Mid., Midventricular.

**Table 4:** Overall segmentation accuracy by slice position.

	Basal mean (±σ)	Midventricular mean (±σ)	Apical mean (±σ)
Precision [%]	80.6	99.7	72.4
Recall [%]	80.6	100.0	86.9
Dice (all slices) [%]	51.7	89.6	43.9
Dice (segmented by both) [%]	75.7 ± 24.9	89.8 ± 8.5	67.6 ± 21.5
Hd [mm]	9.6 ± 9.5	2.9 ± 2.6	4.1 ± 2.9
Abs. vol. diff. (per slice) [ml]	4.2 ± 4.5	0.9 ± 1.3	0.9 ± 0.9

Hd, Hausdorff distance; abs. vol. diff., absolute volume difference.

## 4. Discussion

**Figure 6:**
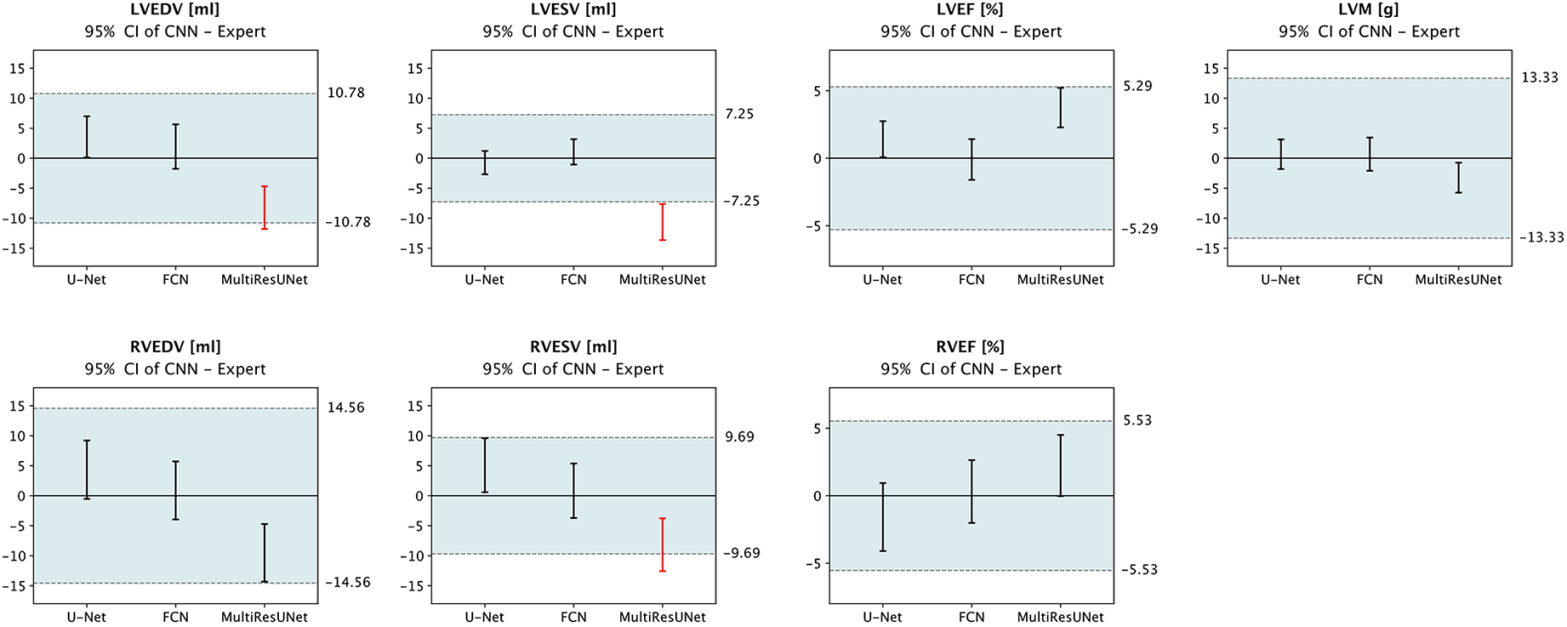
Equivalence testing for clinical parameters. The 95% confidence intervals of mean errors in quantitative clinical parameters are plotted against the tolerance intervals (blue) as defined by Zange et al. (30) and Gröschel et al. (17) based on intraobserver variability. Equivalence is assumed if the respective CI lies completely within the tolerance range. Legend: CI, confidence interval; LVEDV, left ventricular end-diastolic volume; LVESV, left ventricular end-systolic volume; LVEF, left ventricular ejection fraction; LVM, left ventricular myocardial mass; RVEDV, right ventricular end-diastolic volume; RVESV, right ventricular end-systolic volume; RVEF, right ventricular ejection fraction; red, outside tolerance range.

To summarize, our results show that none of the CNN architectures provided a consistent advantage in segmentation quality across different metrics. Segmentation proved more difficult for the right ventricle than for the left; and was more challenging for basal and apical slices than for midventricular slices. When tested for mean differences in clinical parameters, we found that both U-Net and FCN were within predefined published tolerance limits (17, 30) based on intraobserver variability and thus did not show greater deviation from the expert than is acceptable for human readers, whereas the MultiResUNet showed intolerable mean differences for LVEDV, LVESV, and RVESV (Figure 6).

### 4.1. Basal errors cause large volume differences

All CNNs struggled with segmentation decision errors and large volume differences in basal slices, which are sometimes challenging also for experienced physicians. Reasons for this may be, first, the difficulty of a perfect orthogonal slicing during acquisition; second, the partial volume effect as each voxel may contain different structures depending on its slice thickness; and third, a difficult detection of the atrioventricular or semilunar valves. Moreover, segmentation decision of the basal slice can be a matter of definition: a common rule is that at least 50% of the LV blood pool must be surrounded by myocardium (1), which highlights the importance of coherent high-quality training data, so that neural networks can learn such restrictions. The particularly poor results for the RV can be explained by the fact, that it is divided basally by a myocardial invagination into inflow and outflow tract, so that if the valves are not perfectly sliced in-plane, only part of the visible lumen may belong to the ventricle. Basal slices capture large ventricular blood volumes, so errors here weigh heavily.

### 4.2. Segmentation difficulties are not always reflected in clinical parameters

Apical slices showed the lowest Dice values. Reasons for this could be, first, that contrast is frequently impaired due to physiologic apical fat, so that the ventricles cannot always be clearly localized in the short-axis view; and second, that the apex often shows heavy trabeculation. However, the resulting volume differences were no worse than in midventricular slices with excellent segmentation agreement. The simplest explanation is that the heart is narrow at the apex and cardiac structures are small. In addition, for some apical slices at comparable volume, nonoverlapping or only marginally overlapping segmentations were observed, resulting in poor segmentation metrics but small volume differences. Here, segmentation failures also occurred in terms of confusion of left and right ventricles or segmentation of extracardiac structures. Performance on apical slices was comparable for all three models and particularly poor for the LVM.

Besides and specifically in the case of thin LV myocardium, all CNNs predicted displaced segmentations for LVM with little overlap but small area deviation or anatomically implausible, fragmented segmentations. Misinterpretation of large trabeculae or papillary muscles caused mis-segmentation of the ventricular lumen, especially in the thin-walled right ventricle but also in the left ventricle, which in turn led to errors in segmentation of the myocardium. While most of these errors do not have a large impact on overall clinical results, they do affect the trustworthiness of AI models, which is why Bernard et al. also raised a “need for a new metric” (10).

### 4.3. Network architecture may not be the key to achieve the best results

With respect to quantitative clinical parameters, the MultiResUNet showed greater bias compared to U-Net and FCN, which is surprising as publications introducing modifications to CNN architectures usually report their superiority to the unmodified network. However, these findings do not necessarily generalize to other datasets or replicate with different machine learning configurations and pipelines. This becomes evident in the leaderboards of (bio)medical image segmentation challenges, with a comprehensive survey by Litjens et al. noting that “many researchers use the exact same architectures, […] but have widely varying results” (9). Therefore, this study aims to isolate the influence of architectural variations, as opposed to segmentation challenges (10, 22), where complete methodologies with widely varying hyperparameter and training pipeline configurations (including data pre- and postprocessing) were benchmarked.

Against the given background, our results suggest that the more important determinants of network performance are in the variables that were fixed for this comparison. They comprise the dataset used, the configuration of most hyperparameters (e.g., the loss function, learning rate or batch size), and the training pipeline including data pre- and post-processing. This assumption is consistent with findings of Isensee et al. who concluded that “details in method configuration have more impact on performance than do architectural variations” (25); and it is also supported by Baumgartner et al. who found in their comparison of techniques for CMR image segmentation that “the exact architecture played a minor role in the accuracy of the system” (31). The strong influence of data pre-processing and machine learning configuration is further illustrated when looking at the disparate results achieved by participants in the M&Ms Challenge despite their near-universal use of a U-Net architecture as baseline (22).

### 4.4. Data inherent problems and possible solutions

CNN results must be considered in light of human intra- and interobserver variability including possibly inconsistent definitions used in segmentation procedures concerning small trabeculae or basal slices. The fact that most clinical parameters derived from the three CNNs tested were within their tolerance intervals (Figure 6) underscores that the main problem is not with mean deviations but with anatomically implausible segmentation errors, rare outliers, and large basal differences. In (32), slices were automatically classified according to their position and processed by region-specific segmentation CNNs, which improved performance basally and apically. To increase the reliability (13) of deep learning-based models and prevent anatomically implausible segmentations, constraints to preserve cardiac geometry could be implemented *via* a topological loss function (33), shape prior (34) or by automatic correction during post-processing (35). Suinesiaputra et al. (36) found that incorporating landmark and segmentation information from the short-axis and 2- and 4-chamber long-axis views into a combined shape atlas increased robustness basally and apically. The inclusion of spatial and temporal relationships through 3D- or 4D-CNNs using only short-axis view is theoretically attractive, but has so far mostly been inferior in direct comparison (10, 31).

Overall, the uncertainty of deep learning models depends primarily on the data to which they are applied, making generalizability difficult for data characteristics not seen during training (11), which also raises issues for underrepresented entities and complicates a comparison of results outside of standardized settings. For training and test data, the aim should be to achieve heterogeneity of disease entities, patients, scanners, and centers (7, 11), while at the same time ensuring a homogeneous and coherent ground truth annotation. The dataset used in this study, although single-center and single-vendor, comes directly from the clinic and reflects the range of patient populations and clinical indications for CMR. Data augmentation, normalization, as well as network parameter adjustment can help make the best use of limited data to train models, that still generalize well (37). At the same time, data-centric AI may be a suitable approach in small-data settings. However, dealing with outliers against a background of limited training data and extremely low error tolerance in medical diagnostics requires continuous quality control and supervision of all steps in the method pipeline (38, 39).

### 4.5. Automated quantification will increase efficiency and reproducibility

Automated function quantification took a fraction of the time compared to manual analysis and may help address the increasing workload (40) in medical imaging. Results are reproducible, eliminating observer bias, and therefore show promise for increasing reliability and tracking of even small changes in patients over time. In addition, the CNN models can segment all slices of the cardiac cycle to obtain time-volume curves in almost no additional time, prospectively providing extended information for diagnosis. Still, the main obstacle to widespread adoption of automated deep learning based image analysis methods remains their implementation in routine clinical practice (41), as technical, administrative, and regulatory hurdles have not yet been met by an accessible deployment infrastructure.

Our dataset was acquired using Compressed Sensing as an acceleration technique to minimize scan time and duration of breath holds. To date, very limited literature has been published on deep learning methods for image segmentation applied to such data. While the sequence used does not significantly affect diagnostic image quality according to objective criteria, the images have been considered to be blurrier and prone to ghosting artifacts (17), which is why the data presents a particular challenge for image analysis algorithms. Clinical evaluation using the Compressed Sensing sequence was considered equivalent to the standard method in (17). We would expect similar, arguably slightly improved results on a dataset acquired using a standard sequence. The merging of the reconstruction task in Compressed Sensing during image acquisition and the cardiac segmentation task into a joint network could prospectively be advantageous for both (42), which has also been demonstrated for brain MRI (43).

### 4.6. Outlook

To overcome the scarcity of well-labeled and accessible data for training, data sharing platforms (44) as well as technical approaches like multi-view or cross-modal, and semi-, self-, or unsupervised learning (8, 45) offer great potential to accelerate the development of AI. A deployment infrastructure for image analysis methods that integrates with existing workflows is essential to bring AI broadly into the clinic and validate it prospectively (41). Since small structures (e.g., in apical slices, papillary muscles, or thin myocardium) are only a few pixels in size, the training of CNNs is likely to benefit from hyper-resolution. For a readily available semi-automatic solution, segmentation agreement could be estimated without ground truth to flag difficult cases or slices and guide the attention of a supervising expert. In the future, there will be a shift from slice-imaging to volumetric 3D CMR sequences, providing opportunities for new automatic quantification techniques. Segmentation-derived features will be useful for radiomics-based image phenotyping and diagnostic AI (46, 47).

### 4.7. Conclusions

Multilevel analysis allowed for a detailed comparison of differences in quantitative clinical parameters among the three CNNs and their attribution to individual segmentation problems. All three CNNs demonstrated strong correlation to the expert on our dataset, which is primarily explained by low errors in midventricular slices. Segmentation errors clustered in basal and apical slices and are not necessarily reflected in the overall results usually reported. In summary, modifications to CNN architecture might not be the decisive factor in achieving the best results. Our findings further highlight the need for detailed quality assurance of medical AI, as even rare errors that violate medical principles or anatomy can severely undermine confidence in deep learning algorithms. Automatic segmentation combined with fast acquisition will increase the efficiency of cardiac MRI, allowing more patients to benefit from this examination.

### 4.8. Limitations

The focus in defining the three CNNs was on a mostly unaltered reproduction of the published architectures, that all share the same basic network configuration and a similar number of trainable parameters, in favor of which extensive hyperparameter tuning was omitted. The underlying assumption was that the three network architectures are more comparable with similar parameters and network complexity than with individually optimized configurations. Due to the continuous emergence of new CNN architectures, this study does not provide a fully comprehensive comparison of novel architecture variants. Instead, it focuses on an in-depth analysis of recurring problems in cardiac image segmentation with the two most popular and a newer, complex CNN architecture specifically designed to address their weaknesses. To contextualize the qualitative and slice-specific evaluation of the CNNs, an equally nuanced interobserver analysis of human experts may be necessary.

## Data Availability

The datasets presented in this article are not readily available because they contain patient data and cannot be published for legal and privacy reasons. Requests to access the datasets should be directed to JS-M, jeanette.schulz-menger@charite.de.
